# Tau Oligomers Resist Phase Separation

**DOI:** 10.3390/biom15030336

**Published:** 2025-02-26

**Authors:** Lathan Lucas, Phoebe S. Tsoi, Josephine C. Ferreon, Allan Chris M. Ferreon

**Affiliations:** Department of Biochemistry and Molecular Pharmacology, Baylor College of Medicine, Houston, TX 77030, USA; lathanl@bcm.edu (L.L.); phoebe.tsoi@bcm.edu (P.S.T.)

**Keywords:** tauopathy, LLPS, tau oligomers, neurodegeneration, tau pathology

## Abstract

Tau is a microtubule-associated protein that undergoes liquid–liquid phase separation (LLPS) to form condensates under physiological conditions, facilitating microtubule stabilization and intracellular transport. LLPS has also been implicated in pathological Tau aggregation, which contributes to tauopathies such as Alzheimer’s disease. While LLPS is known to promote Tau aggregation, the relationship between Tau’s structural states and its phase separation behavior remains poorly defined. Here, we examine how oligomerization modulates Tau LLPS and uncover key distinctions between monomeric, oligomeric, and amyloidogenic Tau species. Using dynamic light scattering and fluorescence microscopy, we monitored oligomer formation over time and assessed oligomeric Tau’s ability to undergo LLPS. We found that Tau monomers readily phase separate and form condensates. As oligomerization progresses, Tau’s propensity to undergo LLPS diminishes, with oligomers still being able to phase separate, albeit with reduced efficiency. Interestingly, oligomeric Tau is recruited into condensates formed with 0-day-aged Tau, with this recruitment depending on the oligomer state of maturation. Early-stage, Thioflavin T (ThT)-negative oligomers co-localize with 0-day-aged Tau condensates, whereas ThT-positive oligomers resist condensate recruitment entirely. This study highlights a dynamic interplay between Tau LLPS and aggregation, providing insight into how Tau’s structural and oligomeric states influence its pathological and functional roles. These findings underscore the need to further explore LLPS as a likely modulator of Tau pathogenesis and distinct pathogenic oligomers as viable therapeutic targets in tauopathies.

## 1. Introduction

Tau, a protein that binds microtubules in healthy neurons, can become misregulated and dysfunctional in several neurodegenerative diseases, including Alzheimer’s disease (AD) and other tauopathies [1,2,3,4,5,6,7,8,9,10]. In the context of these diseases, Tau transitions from soluble functional monomeric and oligomeric states to assemblies of pathological oligomers and fibrils [11,12,13,14]. While Tau oligomers are often regarded as intermediates on the pathway to fibril formation, growing evidence suggests that some oligomeric species play distinct functional roles in neurons, such as in microtubule binding [12,13,14,15,16,17,18]. In disease, these oligomers act as intermediates on the path to fibril formation, assembling into paired helical filaments (PHFs), which are the primary structural components of neurofibrillary tangles observed in AD and other tauopathies [19,20,21]. Understanding the connection between distinct Tau oligomers and their contributions to pathology will provide critical insight into the mechanisms underpinning the transition of physiological Tau into pathological aggregates.

One phenomenon central to Tau biology is liquid–liquid phase separation (LLPS), which is characterized by the formation of concentrated dynamic condensates composed of soluble Tau [22,23,24,25,26,27]. LLPS is increasingly recognized as a fundamental mechanism for organizing cellular biochemistry [11,28,29,30,31,32,33]. Tau readily undergoes LLPS under physiological conditions, forming condensates shown to play a functional role in stabilizing microtubules in vitro and in neurons [24,34,35,36]. However, Tau LLPS has also been implicated in aggregation pathways, serving as a precursor to amyloid fibril formation [22,37,38,39]. The duality between LLPS supporting normal cellular functions and/or contributing to pathological processes highlights the complex role of LLPS in Tau biology. Similarly, Tau oligomers exhibit functional and pathological properties, making the interactions between oligomerization and LLPS a valuable framework for investigating the interplay between each and their contributions to disease pathology.

In this study, we leveraged dynamic light scattering (DLS) to investigate Tau oligomerization, revealing that the time-dependent transition of monomeric Tau into oligomers results in oligomers that resist LLPS. We show that ThT-negative Tau oligomers, lacking amyloid-like features, integrate into condensates, while larger ThT-positive oligomers, which are amyloidogenic, resist phase separation entirely. Moreover, we found that LLPS promotes the rapid formation of SDS-resistant, heat-stable oligomers. These findings highlight the complex connections between Tau phase separation, oligomerization, and fibrillization.

Our results provide critical insights into the relationship between Tau oligomers and LLPS, highlighting how structural differences in oligomers influence their interaction within condensates, which offers valuable perspectives for targeting tauopathies.

## 2. Materials and Methods

### 2.1. Sample Preparation

Bacterial expression and purification of full-length human wild-type (WT) Tau was performed as previously described [26,40]. Lyophilized Tau protein stocks were dissolved in water and centrifuged at 20,000× *g* for 10 min. Protein samples were filtered twice through a 0.02 µm-filter, and protein concentrations were determined as described below. Purified Tau was run on a gel and stained with Coomassie to evaluate purity.

For fluorescently labeled samples, purified Tau was dye-labeled using N-hydroxysuccinimide (NHS) ester chemistries with Alexa Fluor 488 (A488; Invitrogen, Waltham MA, USA) or Alexa Fluor 647 (A647; Invitrogen). Labeling reactions were conducted at a 1:30 protein:dye ratio and incubated at RT for 1–3 h. Labeled products were purified by reverse-phase HPLC (Agilent, Santa Clara, CA, USA).

### 2.2. Protein Concentration Determination

Concentrations of recombinant Tau were calculated using their UV absorbance extinction coefficients at 280 nm using the Edelhoch method [41,42,43]. Fluorescent protein concentrations were determined using extinction coefficients of the A488 and A647 dyes.

### 2.3. Sample Storage

Monomeric Tau was diluted in de-ionized distilled H_2_O to 20 µM and incubated at RT for aging. DLS was used for each sample preparation to check and ensure that Tau was initially monomeric.

### 2.4. Dynamic Light Scattering Measurements

DLS was performed using the DynaPro Nanostar II (Wyatt Technology, Waters Corporation, Santa Barbara, CA, USA) to measure Brownian motion in solution by analyzing fluctuations in scattered light intensity from a 658 nm-laser. These fluctuations were detected orthogonally and used to calculate the diffusion coefficient (D), which was converted to a hydrodynamic radius (R*_h_*) using the Stokes–Einstein equation

$$R_{h}=\frac{kT}{6\pi \eta D}$$

where k is the Boltzmann constant, T is the absolute temperature, and η is the viscosity of the solution.

Tau samples at 20 µM in water were diluted to 2 µM and loaded into a 45 µL quartz cuvette (Wyatt Technology, Waters Corporation). Measurements were taken at 25 °C with five acquisitions per sample at 2 s intervals over 5 min, repeated in triplicate. Autocorrelation plots were fitted using the Dynamics software version 2.0.1.164 (Wyatt Technology, Waters Corporation) regularization model, and averaged results are reported with compounded standard deviations from three acquisitions and replicates.

### 2.5. Induction of Tau LLPS

Tau that was stored and aged in water was added 1:1 to a 2× LLPS buffer (20% (*v*/*v*) dextran, 10 mM Hepes, pH 8). LLPS samples were thoroughly mixed and added directly to coverslips or to a gel loading buffer for imaging or SDS-PAGE, respectively.

### 2.6. Transmission Light Microscopy

Tau samples were transferred onto the coverslip of a 35 mm μ-Dish (ibidi, Martinsried, Germany). Brightfield images were captured using the EVOS FL Imaging System (Invitrogen). All images were taken at 80% white light power.

### 2.7. ThT Staining Microscopy

Tau samples were transferred onto the coverslip of a 35 mm μ-Dish (ibidi). Samples containing ThT were imaged using the EVOS FL Imaging System (Invitrogen) equipped with a GFP excitation LED light and filter. All images were taken at 72% light power.

### 2.8. Purification of Tau Oligomers from Aged Samples

Aged Tau samples were spun down at 20,000× *g* for 30 min at 4 °C. The supernatant was removed and the pellet was resuspended in 100 µL de-ionized distilled H_2_O. Resuspended Tau oligomers were passed through a 100 kDa molecular weight cut off Amicon Ultra—0.5 mL (Millipore, Burlington, MA, USA) concentrator until <10 µL remained above the filter. An amount of 90 µL of de-ionized distilled H_2_O was added, and the sample was again passed through the concentrator until 10 µL remained. The remaining 10 µL was collected, and the Tau oligomer concentration was determined using methods described above.

### 2.9. Spinning Disk Fluorescent Microscopy

Mixtures of fluorescently labeled monomeric (A488) and oligomeric (A647) Tau samples undergoing LLPS (3 µM ThT, 10% (*v*/*v*) dextran, 10 µM Tau, 5 mM Hepes, pH 8) were mixed and transferred onto the coverslip of a 35 mm μ-Dish (ibidi). Samples were imaged using the Nikon Eclipse Ti2 (Nikon, Japan) equipped with a CSU-W1 Confocal Scanner Unit (Yokogawa, Japan) for spinning disk confocal microscopy using a 40× objective. Images were taken in Z-stacks (30 µm thick), and 3D image rendering and droplet quantification was performed using the NIS-Elements software version 5.41.01 (Nikon, Tokyo, Japan).

### 2.10. Oligomer Seeding Assay

An amount of 1 µM purified oligomers from 5-day- and 3-week-aged Tau samples were incubated with or without 70 µM fresh monomeric Tau and 3 µM ThT in water. Samples were prepared in triplicate in a round bottom 384-well plate (Invitrogen), and ThT fluorescence was recorded every 30 min for 22 h at 37 °C using a Spark Multimode Microplate Reader (Tecan, Männedorf, Switzerland). Fluorescence values were background-corrected by subtracting the average signal from corresponding oligomer/ThT-only or ThT-only controls and were baseline-corrected.

### 2.11. Tau Oligomer Visualization via SDS-PAGE

Samples of Tau aged various times were either induced to undergo LLPS (10% (*v*/*v*) dextran, 5 mM Hepes, pH 8) or retained in water and incubated at RT for 30 min. Following incubation, samples were mixed 1:1 (*v*/*v*) with a 2× SDS loading buffer supplemented with 10 mM DTT and 5 mM TCEP (1× = 2% (*w*/*v*) SDS, 2 mM DTT, 10% (*v*/*v*) glycerol, 12.5 mg bromophenol blue, 20 mM Tris, pH 7). Samples were heated at 95 °C for 5 min before loading onto a 4–20% Criterion TGX Precast Gel (Bio-Rad, Hercules, CA, USA). Electrophoresis was performed at 225 V for ~40 min in Tris/Glycine/SDS running buffer (VWR, Radnor, PA, USA). Gels were fixed in 7.5% acetic acid for 30 min, stained with 1X Sypro Orange (Invitrogen) for 30–60 min and briefly destained with 7.5% acetic acid for 20 sec before imaging (excitation: 530 ± 28 nm; emission: 605 ± 50 nm) using a ChemiDoc MP Imaging System (Bio-Rad).

### 2.12. Statistical Analysis

Unless otherwise stated in the figure legends, ANOVA multiple comparisons were employed for direct comparisons between samples. All data points (DLS, microscopy and gels) were collected in three independent replicates. All DLS graphs show the average and compounded standard deviation (SD), and all bar and line graphs show the average and standard error of measurement (SEM). Regardless of the analysis method, significance levels were denoted by *, **, ***, and **** for *p* < 0.05, *p* < 0.01, *p* < 0.001, and *p* < 0.0001. Graphs were generated using Prism v. 10.3.0 (GraphPad, San Diego, CA, USA).

## 3. Results

### 3.1. Tau Forms Oligomers over Time

Tau belongs to the MAP2/Tau family of structurally similar microtubule-associated proteins consisting of MAP2, MAP4 and Tau [2]. A full-length human Tau (2N4R), consisting of two N-terminal inserts (2N) and four microtubule-binding repeats (4R), represents the largest isoform of Tau in the human brain excluding “big Tau” [9,44]. Notably, 2N4R Tau monomers rapidly form dimers and oligomers within hours in the presence of negatively charged cofactors such as heparin [45,46]. In the absence of cofactors, oligomerization occurs more slowly, requiring several days to generate stable oligomers, but it can be accelerated as a function of temperature between 27 °C and 40 °C [46,47].

**Figure 1:**
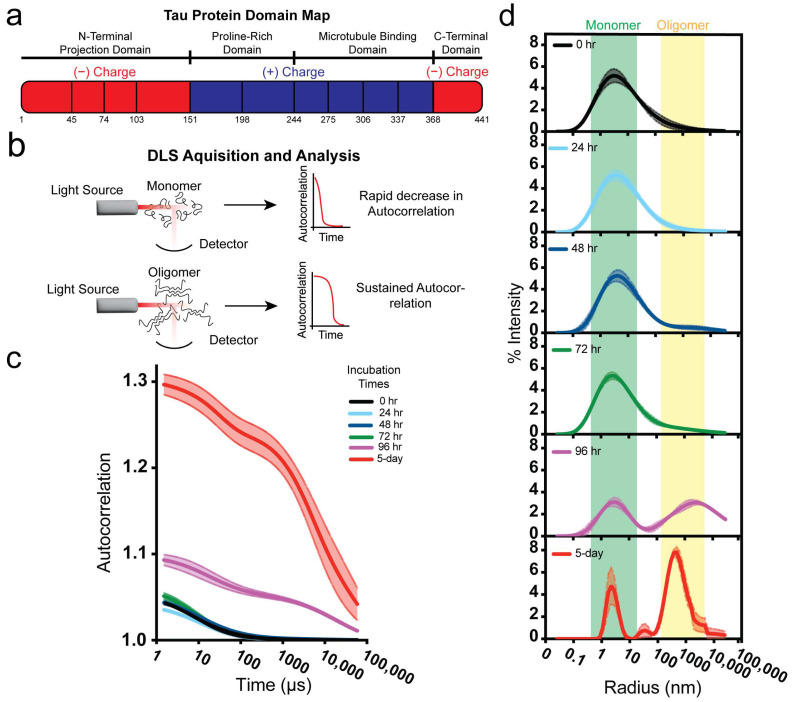
Tau oligomerization monitored by dynamic light scattering over time. (**a**) The Tau primary structure schematic illustrates a negatively charged N-terminal domain, positively charged proline-rich and microtubule-binding domains and a negatively charged C-terminal domain. (**b**) Detection of protein oligomerization state by DLS. (**c**) The DLS auto-attenuation data of differentially aged 20 µM Tau in water, which was diluted to 2 µM for analysis. (**d**) Size distributions and populations of differentially aged Tau samples.

Using dynamic light scattering (DLS), we monitored Tau oligomerization kinetics in water at room temperature, allowing us to observe oligomeric species (Figure 1). On the basis of light scattering patterns, freshly prepared monomeric Tau (20 µM) remained primarily monomeric for up to 72 h, with detectible oligomerization starting as early as 48 h.

After 96 h, DLS autocorrelation analysis showed the emergence of oligomers, which became more abundant at 120 h (5 days; Figure 1c). Hydrodynamic radius measurements revealed distinct distributions for monomeric (~7 nm) and oligomeric (~750 nm) Tau species. These findings establish the clear emergence of Tau oligomers after 120 h (5 days) under these conditions.

### 3.2. Tau Oligomerization Reduces Condensate Abundance

**Figure 2:**
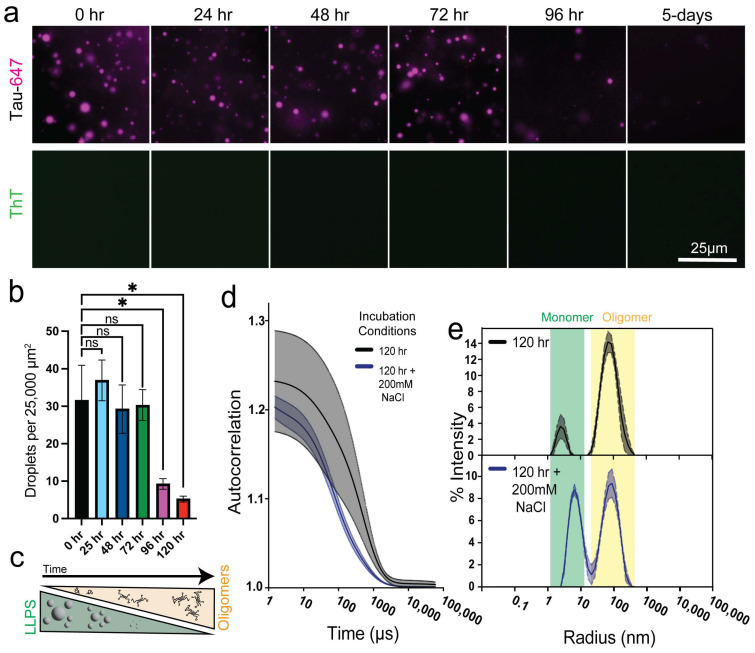
ThT-negative Tau oligomers resist LLPS. (**a**) Fluorescence microscopy of 10 µM Tau of various ages in 3 µM ThT, 10% (*v*/*v*) dextran, 5 mM Hepes, pH 8. (**b**) Quantification of droplet abundance at the bottom of the plate; *n* = 3, error bars = SEM. *, and ns represent *p* ≤ 0.05 and not significant, respectively. (**c**) Model depicting the relationship between Tau oligomer abundance and LLPS. (**d**) DLS auto-attenuation data of 2 µM Tau aged in water for 120 h treated with and without 200 mM NaCl. (**e**) Size distributions, derived from D, of the 120 h aged Tau (top panel) and the 120 h aged Tau treated with 200 mM NaCl (bottom panel).

After characterizing Tau oligomerization kinetics at 20 µM in water, we assessed the impact of oligomerization on LLPS. Tau aged at various time points was diluted to 10 µM in 5 mM Hepes, pH 8, with 10% (*v*/*v*) dextran to induce LLPS. Thioflavin T (ThT, 3 µM) was added to detect amyloid-like structures. Freshly prepared monomeric Tau readily underwent LLPS and was ThT-negative (Figure 2a; Supplemental Figure S2a). Tau aged for at least 96 h exhibited a reduced propensity for LLPS, while samples aged for 5 days displayed the fewest LLPS droplets (Figure 2a,b; Supplemental Figure S2b). Notably, all Tau oligomers that were formed within 5 days remained ThT-negative, even under conditions promoting LLPS (Figure 2a; Supplemental Figure S2a). These findings indicate that, as Tau oligomerized over time, its ability to undergo LLPS diminished (Figure 2a–c).

Given that Tau dimerization and LLPS are driven by electrostatic interactions [48,49,50], we investigated whether early-stage oligomers resistant to LLPS are also electrostatically stabilized. We first observed the effect of NaCl on Tau LLPS at each time point. 200 mM NaCl prevented condensate formation regardless of the oligomerization state (Supplemental Figure S3). We next tested the effect of NaCl on oligomer stabilization. A sample of 20 µM Tau aged in water for 120 h was confirmed to contain oligomers via DLS (Figure 2d). The addition of 200 mM NaCl to disrupt electrostatic interactions resulted in significant depletion of DLS autocorrelation signals on the longer time scales, suggesting de-population in oligomeric species (Figure 2d). Nevertheless, the average hydrodynamic radius of both oligomer populations in the presence and absence of salt remained the same (Figure 2e). Tau samples further aged for 240 h displayed similar de-population of the oligomeric species in salt (Supplemental Figure S2c,d). Taken together, these results indicate differential properties and stabilities among similarly sized Tau oligomers.

### 3.3. ThT-Positive Tau Oligomers Spontaneously Form After Extended Incubation

**Figure 3:**
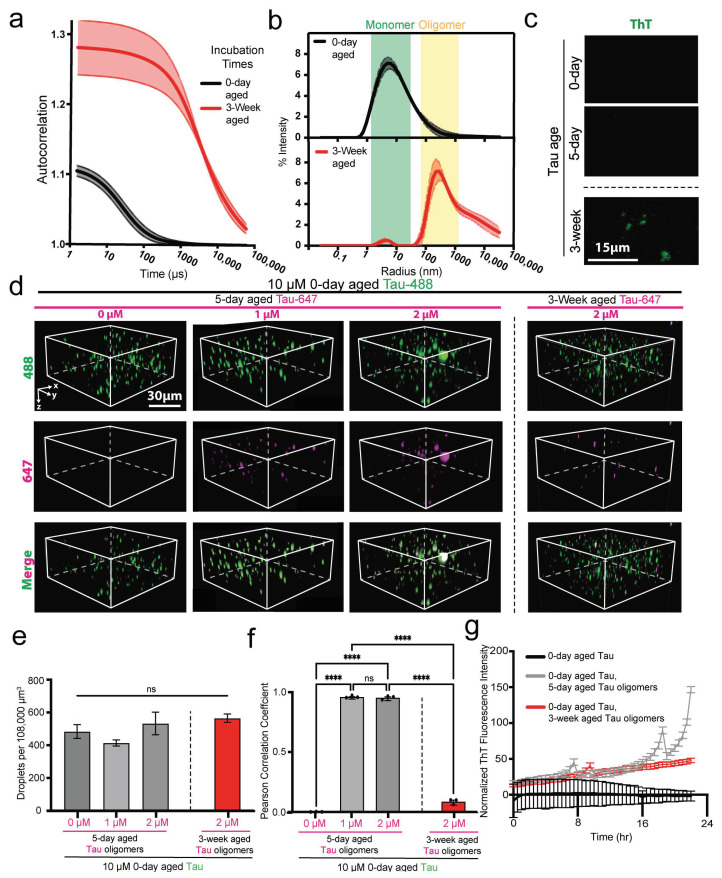
ThT-positive Tau oligomers disfavor condensate recruitment. (**a**) DLS auto-attenuation data of 2 µM 0-day- and 3-week-aged Tau samples. (**b**) Size distributions of 0-day (top panel) and 3-week (bottom panel)-aged Tau. (**c**) Fluorescence microscopy of 10 µM Tau (0-day, 5-day, or 3-week-aged) in 3 µM ThT, 10% (*v*/*v*) dextran, 5 mM Hepes, pH 8. (**d**) Three-dimnsional spinning-disk confocal microscopy images (120 µm × 120 µm × 60 µm) of 10 µM 0-day Tau mixed with different concentrations of pure oligomers derived from either 5-day- or 3-week-aged Tau samples. (**e**) Quantification of droplet abundance within the entire 120 µm × 120 µm × 60 µm region described in D; *n* = 3, error bars = SEM. ns represents not significant. (**f**) Pearson correlation coefficient analysis of the spinning disk microscopy images quantifying high co-localization (coefficient = 1) or low co-localization (coefficient = 0) between monomeric and oligomeric Tau; *n* = 3, error bars = SEM. ****, and ns represent *p* ≤ 0.0001 and not significant, respectively. (**g**) Oligomer seeding kinetics assay comparing ThT (3 µM) fluorescence intensity of mixtures containing 70 µM monomeric Tau with and without 1 µM oligomers from 5-day- and 3-week-aged samples; *n* = 3, error bars = SEM.

To generate late-stage Tau oligomers, 20 µM Tau was aged in water for 3 weeks, and DLS was employed to assess the oligomer size. Compared to 0-day-aged monomeric Tau, 3-week-aged Tau exhibited autocorrelation signals at longer time scales, indicating the formation of high-molecular-weight (HMW) oligomers (Figure 3a). Hydrodynamic radius measurements of 0-day Tau samples confirmed the presence of distinct monomers (~7 nm), which were nearly completely depleted in 3-week-aged samples.

In contrast, 3-week-aged samples displayed a broad distribution of oligomers, ranging from ~750 to >1000 nm (Figure 3b). LLPS induction and ThT fluorescence staining of 10 µM 0-day-, 5-day-, and 3-week-aged Tau samples revealed that only 3-week-aged Tau formed ThT-positive aggregates (Figure 3c). These findings demonstrate that late-stage Tau oligomers are larger and exhibit amyloidogenic properties.

### 3.4. Early Tau Oligomers Are Recruited into Condensates

We tested whether Tau oligomers of different ages could be recruited into condensates by mixing them with phase-separation-competent Tau derived from 0-day-aged monomeric samples. We purified fluorescently labeled early-stage ThT-negative oligomers from 5-day-aged samples and late-stage ThT-positive oligomers from 3-week-aged samples and confirmed the absence of the monomeric species using DLS (Supplemental Figure S4). Adding either early-stage or late-stage oligomers to 10 µM 0-day-aged Tau under LLPS-inducing conditions did not significantly change the abundance of condensates (Figure 3d,e), showing that 0-day-aged Tau readily forms condensates even in the presence of various oligomeric species.

Early-stage oligomers from 5-day-aged samples were recruited into and colocalized within Tau condensates, as evidenced by the high Pearson correlation coefficient between the fluorescence signals of monomeric and oligomeric Tau (Figure 3f). In contrast, late-stage oligomers from 3-week-aged samples exhibited a low Pearson correlation coefficient, indicating that late-stage oligomers were not recruited into condensates (Figure 3f). To assess the seeding capacity of oligomers derived from 5-day- and 3-week-aged samples, we monitored the kinetics of Tau oligomerization using ThT fluorescence at 37 °C. Monomeric Tau (70 µM) was incubated alone or in the presence of oligomers (1 µM) from either 5-day- or 3-week-aged samples. Both oligomeric species significantly enhanced ThT fluorescence intensity compared to monomeric Tau alone (Figure 3g). Notably, oligomers from 5-day-aged samples induced a greater increase in ThT fluorescence, suggesting that the resulting aggregates exhibit a higher ThT binding affinity and may be structurally distinct from those seeded by 3-week-aged oligomers (Figure 3g). Altogether, our data suggest that, while early-stage oligomers can interact with and integrate into Tau condensates, late-stage amyloidogenic oligomers remain excluded, while both oligomeric species can seed monomeric Tau oligomerization.

### 3.5. Heat-Stable, SDS-Resistant Oligomers Rapidly Form in Condensates

**Figure 4:**
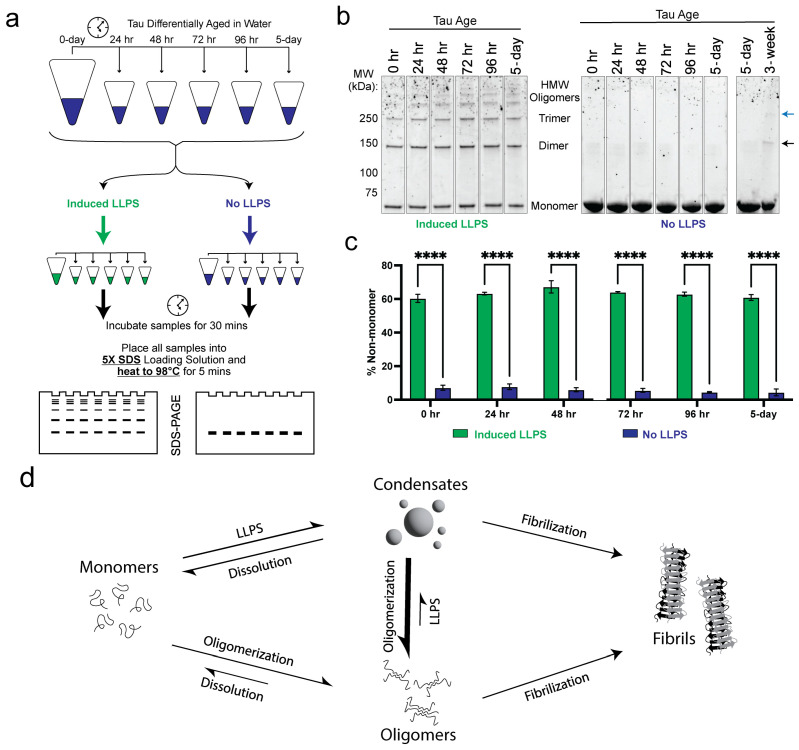
Tau LLPS rapidly induces stable oligomer formation. (**a**) Diagram depicting the experimental approach used to assess SDS-resistant and 98 °C stable Tau oligomer formation with and without LLPS. (**b**) SDS-PAGE of differentially aged Tau samples in water that were mixed (left) or otherwise (right) with LLPS buffer (10% (*v*/*v*) dextran, 5 mM Hepes, pH 8) prior to gel loading. Total protein was detected with Sypro Orange. Stable dimers and timers are indicated by the black and blue arrows, respectively. (**c**) Quantification of Tau species larger than monomer from SDS-PAGE; *n* = 3, error bars = SEM. **** represents *p* ≤ 0.0001. (**d**) Model depicting the interplay between Tau phase separation, oligomerization and fibrilization.

To investigate how LLPS influences Tau oligomerization, we compared the stability of Tau oligomers that were aged under two conditions: maintained exclusively in water or subjected to LLPS after aging (Figure 4a). Remarkably, 0- to 5-day-aged Tau samples, when mixed into LLPS-inducing conditions, rapidly formed heat-stable and SDS-resistant oligomers (Figure 4a,b; Supplemental Figure S5). In contrast, Tau samples retained exclusively in water did not produce heat-stable or SDS-resistant species during the same timeframe (Figure 4b; Supplemental Figure S5). Quantification of oligomeric species across all Tau samples aged up to five days revealed that these stable oligomers formed exclusively within condensates (Figure 4b,c; Supplemental Figure S5).

Interestingly, after 3 weeks of aging in water, Tau formed heat-stable and SDS-resistant oligomers even without undergoing LLPS, indicating that extended aging can also produce stable oligomeric species (Figure 4b; Supplemental Figure S5). These findings suggest that LLPS significantly accelerates oligomerization kinetics and enhances the stability of early-stage oligomers, while prolonged aging independently drives the formation of stable oligomers. We show that oligomeric Tau resists LLPS but that early-stage oligomers are recruited into condensates (while late-stage amyloidal oligomers are not) (Figure 4d). We also show that Tau LLPS creates an environment conducive to the rapid formation of stable oligomeric species (Figure 4d).

## 4. Discussion and Conclusions

Tau oligomerization and fibril formation are central to the progression of neurodegenerative tauopathies, driving toxic protein deposition and cellular dysfunction [1,2,3,4,5,6,7,8,9,10,11,12,13,14]. LLPS has emerged as a key mechanism in Tau biology, linking the formation of dynamic condensates to both functional and pathological oligomer formation [22,28,29,30,31,32,33,37,38,39,51]. While Tau LLPS and oligomerization are known to be interconnected, the precise impact these processes have on each other remains unexplored. Understanding this relationship reveals insights into how distinct oligomeric species relate to LLPS and how oligomerization and phase separation link to protein aggregation pathways.

Our results demonstrate that not all Tau oligomers are created equal. Early-stage ThT-negative oligomers retain sufficient structural flexibility to interact with and integrate into condensates. In contrast, late-stage ThT-positive oligomers resist LLPS entirely, reflecting a structural rigidity that is incompatible with phase separation. This differential behavior emphasizes that oligomeric species exhibit distinct physico-chemical properties, influencing their aggregation and LLPS potential. Furthermore, our findings demonstrate that oligomers formed within LLPS environments after incubation (30 min) were SDS-resistant and heat-stable yet remained ThT-negative, indicating that these oligomers are distinct from those aged in physiological buffer and are likely stabilized by mechanisms dependent on the condensate microenvironment.

Tau LLPS can serve as a regulatory mechanism in neuronal biology, providing a controlled environment to modulate Tau’s aggregation-prone nature. In LLPS-favoring conditions, monomeric Tau forms stabilized oligomers capable of participating in physiological functions, such as microtubule stabilization [24,34,35,36]. The dynamic nature of LLPS enables cells to regulate local Tau concentrations through condensate formation and dissolution, allowing for a nuanced handle on Tau localization and function. Moreover, the dynamic equilibrium between Tau monomers, oligomers, and condensates prevents prolonged condensate aging that can lead to toxic pathological aggregation. Investigating how different Tau oligomeric species influence LLPS and aggregation pathways may reveal key regulators that shift the balance between functional and pathological Tau states.

## Data Availability

Source data for plots, raw data for counts and intensity measurements, and uncropped gel images generated in this study were submitted to the journal and are available from the corresponding authors upon request.

## Supplementary Materials

The following supporting information can be downloaded at: https://www.mdpi.com/article/10.3390/biom15030336/s1. Figure S1: Coomassie-stained SDS-PAGE of overloaded (~6.5 µg) purified recombinant human 2N4R Tau. Figure S2: ThT-negative Tau oligomers resist LLPS. Figure S3: Fluorescence microscopy of 10 µM Tau of various ages in 3 µM ThT, 10% (*v*/*v*) dextran, 200 mM NaCl, 5 mM Hepes, pH 8. Figure S4: Size distributions of Tau oligomer purified from samples aged for 5 days and 3 weeks. Figure S5: Uncropped SDS-PAGE gels.
