# Supplementary material for: Tau Oligomers Resist Phase Separation
Source: Biomolecules. 2025 Feb 26;15(3):336. doi: 10.3390/biom15030336 (PMC11940599; doi:10.3390/biom15030336)
Supplement: Supplementary file 1 [file biomolecules-15-00336-s001.zip › biomolecules-3455315-supplementary.pdf]

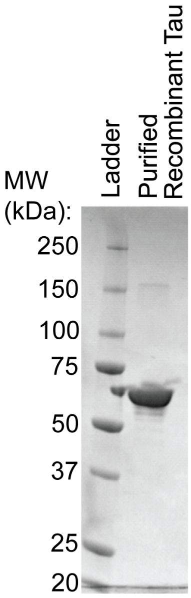

Figure S1. Coomassie-stained SDS-PAGE of overloaded (~6.5 µg) purified recombinant human 2N4R Tau.

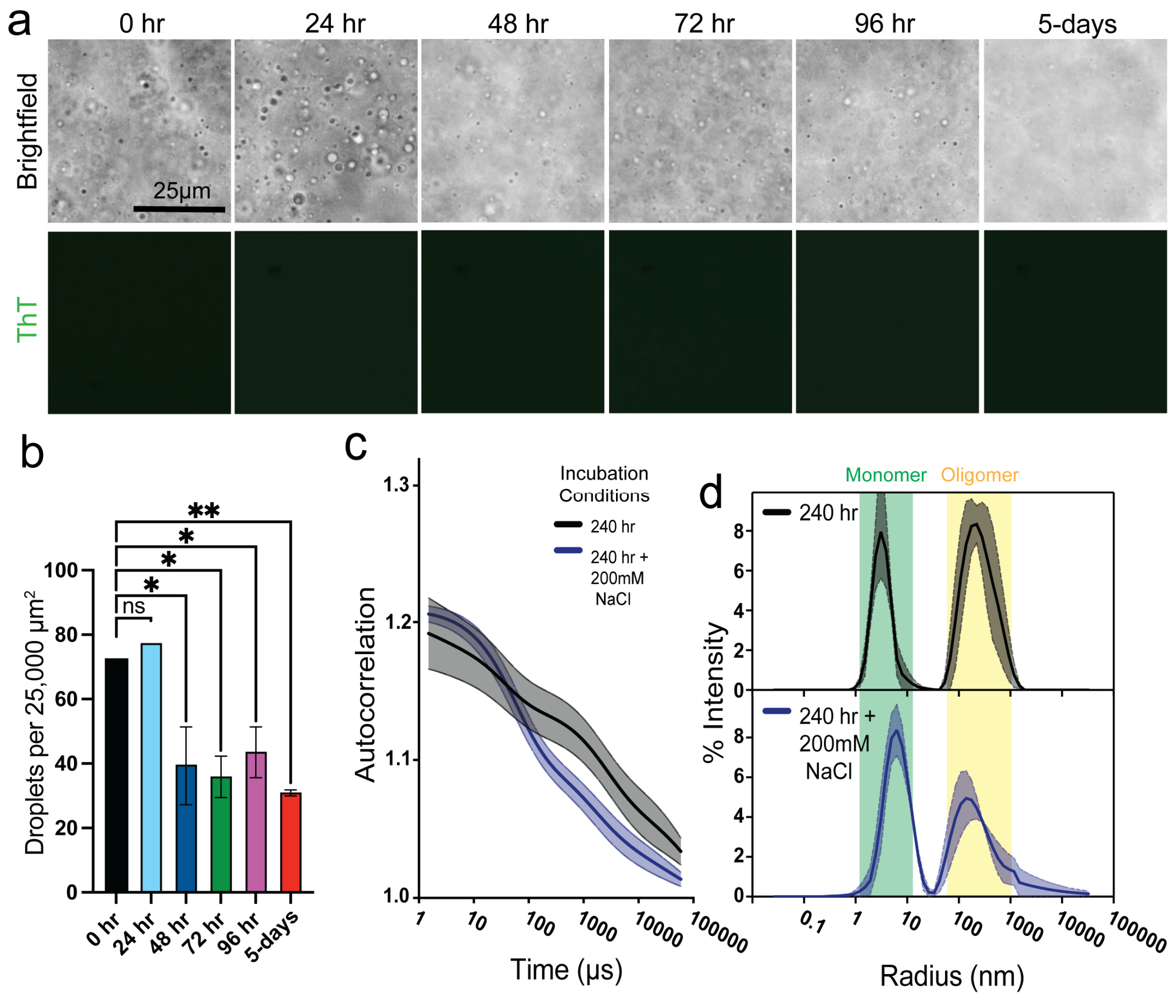

Figure S2. ThT-negative Tau oligomers resist LLPS. **a.** Brightfield and Fluorescence microscopy of 10 µM Tau of various ages in 3 µM ThT, 10% (*v*/*v*) dextran, 5 mM Hepes, pH 8. **b.** Quantification of droplet abundance at the bottom of the plate; $n = 3$, error bars = SEM. **c.** DLS auto-attenuation data

of 2 µM Tau aged in water for 240 hr treated with and without 200 mM NaCl. **d.** Size distributions, derived from C., of the 240 hr aged Tau (top panel) treated with 200 mM NaCl (bottom panel).

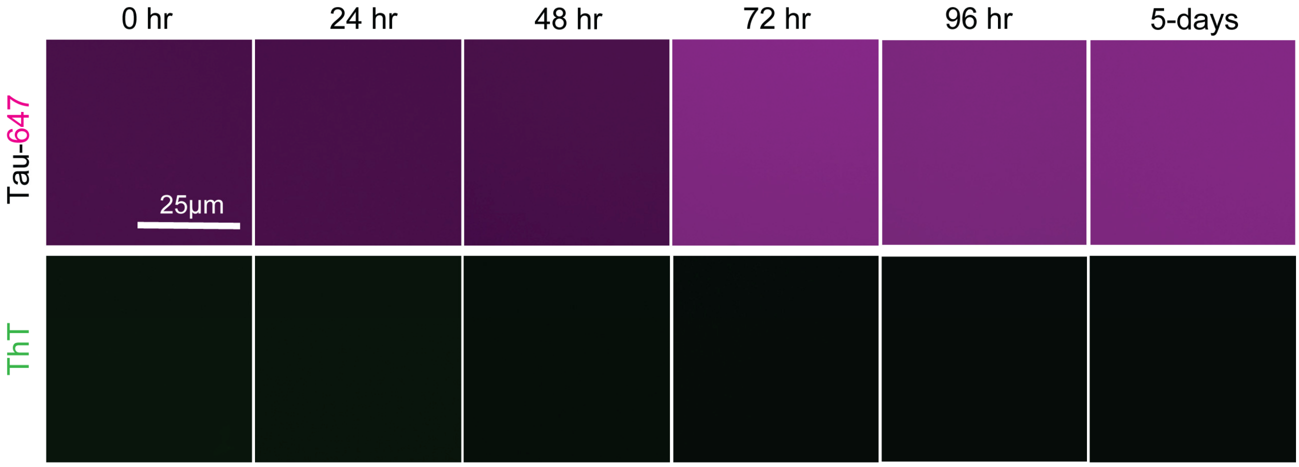

Figure S3. Fluorescence microscopy of 10 µM Tau of various ages in 3 µM ThT, 10% (*v*/*v*) dextran, 200mM NaCl, 5 mM Hepes, pH 8.

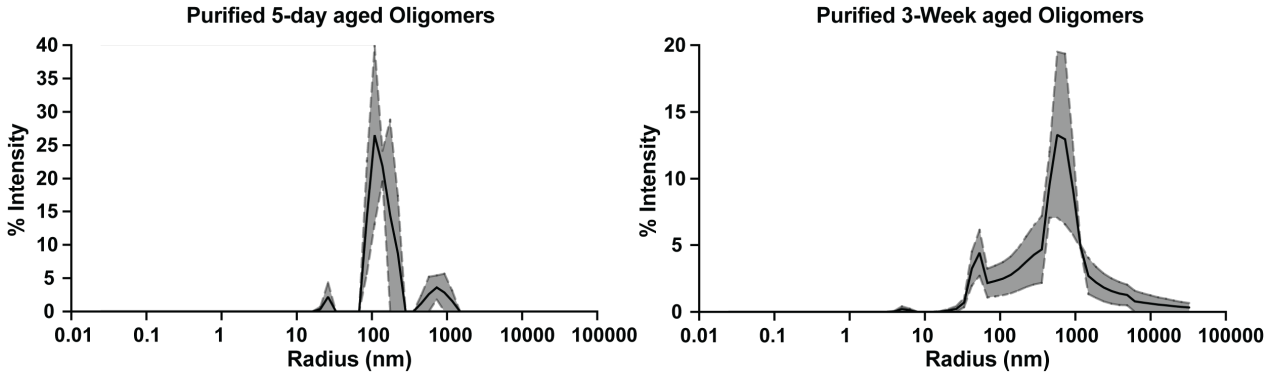

Figure S4. Size distributions of Tau oligomers purified from samples aged for 5 days and 3 weeks.

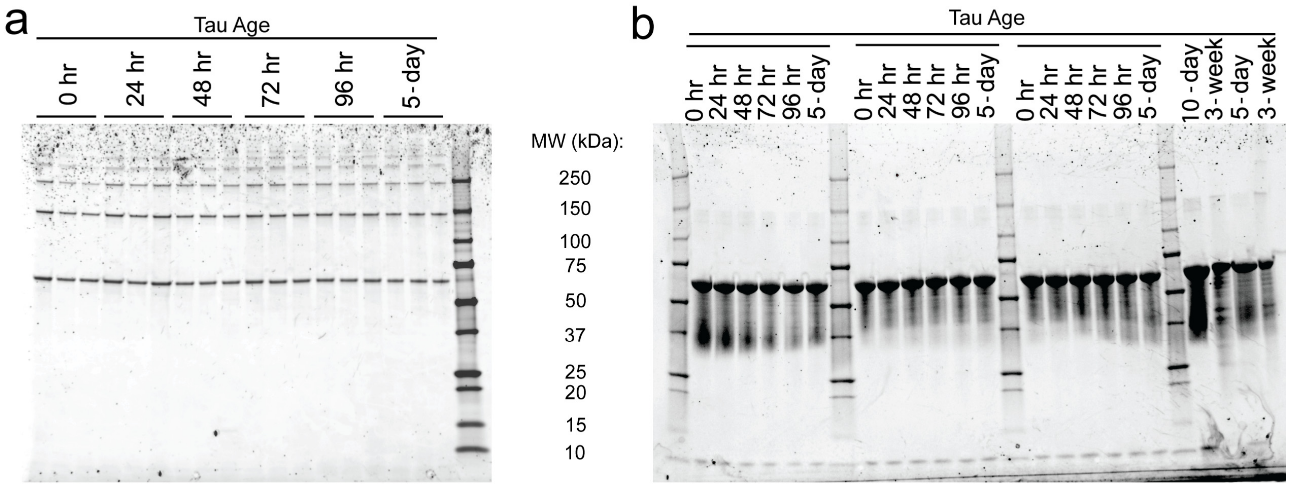

Figure S5. Uncropped SDS-PAGE gels. **a.** SDS-PAGE of Tau samples differentially aged in water and induced to undergo LLPS for 30 min with LLPS buffer (10% (*v*/*v*) dextran, 5 mM Hepes, pH 8) prior to gel loading. **b.** SDS-PAGE of differentially aged Tau samples stored in water without undergoing LLPS prior to gel loading.
